# Epidemiological and economic burden of potentially HPV-related cancers in France

**DOI:** 10.1371/journal.pone.0202564

**Published:** 2018-09-20

**Authors:** Laurent Abramowitz, Jean Lacau Saint Guily, Micheline Moyal-Barracco, Christine Bergeron, Hélène Borne, André Dahlab, Xavier Bresse, Mathieu Uhart, Charlotte Cancalon, Laura Catella, Stève Bénard

**Affiliations:** 1 Service d’hépato-gastroentérologie et proctologie, Hôpital Bichat-Claude Bernard, Paris, France; Ramsay Général de Santé, Clinique Blomet, Paris, France; 2 Service d'ORL et Chirurgie Cervico-Faciale, Hôpital Tenon, Sorbonne Université, Paris, France; 3 Dermatologist, Paris, France; 4 Head of the Pathology Department Laboratoire-Cerba, Cergy-Pontoise, France; 5 Gynecologist, Paris, France; 6 MSD Vaccins, Lyon, France; 7 Health Economist, Lyon, France; 8 Stève Consultants, Oullins, France; Laboratoire National de Santé, LUXEMBOURG

## Abstract

Human papillomaviruses (HPV) infection is now known to be responsible for almost all cervical cancers, and for a substantial fraction of Head and Neck cancers (HNCs). However, comprehensive epidemiological and economic data is lacking in France, especially for rarer potentially HPV-related cancers, which include anal, vulvar and vaginal cancers. Using the national comprehensive database of French public and private hospital information (PMSI), we assessed prevalence and incidence of patients with in-hospital diagnosis for potentially HPV-related cancers in 2013, and estimated costs related to their management over a 3-year period after diagnosis in France. Concerning female genital cancers, 7,597, 1,491 and 748 women were hospitalized for cervical, vulvar and vaginal cancer in 2013, respectively, with 3,120, 522 and 323 of them being new cases. A total of 4,153 patients were hospitalized for anal cancer in 2013, including 1,661 new cases. For HNCs, 8,794 and 14,730 patients were hospitalized for oral and oropharyngeal cancer in 2013, respectively; 3,619 and 6,808 were new cases. Within the 3 years after cancer diagnosis, the average cost of hospital care per patient varied from €28 K for anal cancer to €41 K for oral cancer. Most expenditures were related to hospital care, before outpatient care and disability allowance; they were concentrated in the first year of care. The total economic burden associated with HPV-potentially related cancers was about €511 M for the French National Health Insurance over a 3 years period (2011 to 2013), ranging from €8 M for vaginal cancer to €222 M for oropharyngeal cancer. This study reported the most up-to-date epidemiological and economic data on potentially HPV-related cancers in France. These results may be used to evaluate the potential impact of new preventive strategies, namely the generalized organized screening of cervical cancer and the nine-valent HPV vaccine, indicated in the prevention of cervical, vaginal, vulvar and anal cancers.

## Introduction

Human papillomaviruses (HPV) infection is known to be responsible for various diseases including precancerous lesions, which may progress towards cancer within 5 to 20 years [1].

Most common HPV-related cancers are represented by female genital cancers—including cervical, vulvar and vaginal cancers -, anal cancers, and head and neck cancers (HNCs). Among genital cancers, the primary HPV-related cancer is cervical cancer; since HPV is a key agent for their development almost all cervical cancers would be related to HPV, genotypes 16/18/31/33/45/52/58 being responsible for up to 92% of cervical cancers [2–5]. HPV is also related to a significant proportion of vaginal and vulvar cancers, with 71% and 19% being related to HPV in Europe, respectively [6,7]. Anal cancers have found to be HPV-related in 88% to 97% of cases in Europe; 78% of anal cancers are related to HPV genotypes 16/18 [5,8,9]. The prevalence of HPV in HNCs has been estimated at 46.5% for oropharyngeal cancers and 10.5% for oral cavity cancers in France, with HPV genotype 16 being responsible for 89.7% and 95.5% of HPV-related oropharyngeal and oral cancers, respectively [10]. This prevalence was found to range between 22% and 47% for oropharyngeal cancers and between 8% and 11% for oral cancers in Europe [11].

The epidemiology of potentially HPV-related cancers is well documented in the literature for most common cancers, including cervical cancers and HNCs [2–4]. On the opposite, data remain very sparse and dated for less frequent cancers, including anal, vaginal and vulvar cancers and, to our knowledge, do not exist for the French population [7,12]. In this context, and given the rapid rise of potentially HPV-related cancer incidence [13–15], there is a need for a more comprehensive and recent description of these cancers. In this study based on real world practice, we took advantage of the comprehensive data of the French public and private hospital discharge database (*Programme de Médicalisation des Systèmes d’Information*, PMSI) (i) to report epidemiological data of the most frequent cancers potentially related to HPV in hospitalized patients in France and (ii) to describe long-term total direct medical costs related to the management of these cancers. In addition, based on Population Attributable Fractions (PAFs) available from the literature, a complementary analysis has been conducted to estimate epidemiological data and costs associated to cancers attributable to HPV.

## Materials and methods

Study methods have been validated by a multidisciplinary qualified independent scientific committee (ISC).

### Study design

#### Epidemiological analysis

The epidemiological analysis consisted of the assessment of (i) prevalence of potentially HPV-related cancers in hospitalized patients through a cross-sectional analysis of PMSI data in France and (ii) their incidence (*i*.*e*. number of new cases) in hospitalized patients based on a longitudinal analysis of PMSI data. Both prevalence and incidence were estimated for three years studied: 2011 (*i*.*e*. 1 January 2011 to 31 December 2011), 2012 and 2013.

#### Economic analysis

After describing the patterns of therapeutic management of potentially HPV-related cancers, a descriptive economic analysis has been performed on the incident population of patients with a first in-hospital diagnosis of potentially HPV-related cancers in 2011. We considered total direct medical costs related to cancer care management over a 3 years’ follow-up period after cancer diagnosis, from the French National Health Insurance perspective.

### Source of data: The PMSI database

The PMSI database is a national and comprehensive database of all hospital discharges in French health care facilities, including public and private hospitals [16,17]. It provides information on hospitalized patients, notably demographic (*e*.*g*. sex, age, gender, department and region of residence) and medical data, this later including dates of start and end of stays, length of stay, reasons for hospital admission (*i*.*e*. discharge diagnosis), medical unit(s) of stays, medical procedures performed during the stay, costly drugs dispensed during the stay, and, where applicable, hospital inpatient date of death. It also contains economic information for each hospital stay [18]. All information concerning a same patient is linked together using a unique identifier. Discharge diagnoses are coded in the PMSI using the International Classification of Diseases (ICD-10) [19]. They are entered as primary (main reason for admission), related (disease context of the primary diagnosis) and associated (related to complications and other comorbidities) diagnoses for each stay [16,17]. Medical procedures are coded using the national Common Classification of Medical Acts (*Classification commune des actes médicaux*, CCAM).

### Selection criteria and study populations

Studied potentially HPV-related cancers focused on most common cancers in France, *i*.*e*. with at least 100 observed new cases per year [20,21]. Patients were selected within the PMSI according to a list of ICD-10 codes corresponding to codes of cancers for which at least a fraction is known to be related to HPV (*i*.*e*. potentially HPV-related cancers) from the literature (Table 1). ICD-10 codes could be encoded as primary, related or associated diagnosis for hospital stays during the study period (2011, January 1 to 2013, December 31). Three anatomical sites of potentially HPV-related cancers of interest were considered for the present study: (i) female genital cancers, including cervical, vaginal and vulvar cancers, (ii) anal cancers, and (iii) HNCs, including oral cancer, oropharyngeal cancer, and unspecified oral and/or oropharyngeal cancers, *i*.*e*. for which classification into oral or oropharyngeal group was undetermined based on PMSI data. All ICD-10 coding algorithms for the identification of study populations and selection criteria have been validated by the ISC.

**Table 1:** List of ICD-10 codes selected for the identification of potentially HPV-related cancers.

Cancer anatomical site	Cancer location	ICD-10 codes
**Female genital cancer**	**cervical cancer**	C53: Malignant neoplasm of cervix uteri (C53.0, C53.1, C53.9)
	**vulvar cancer**	C51: Malignant neoplasm of vulva (C51.0, C51.1, C51.2, C51.9)
	**vaginal cancer**	C52: Malignant neoplasm of vagina
**Anal cancer**	**anal cancer**	C21: Malignant neoplasm of anus and anal canal (C21.0, C21.1, C21.2)
**Head and neck cancers (HNCs)**	**oral cancer**	C00: Malignant neoplasm of lip (C00*)
		C02: Malignant neoplasm of other and unspecified parts of tongue (C02.0, C02.1, C02.2, C02.3)
		C03: Malignant neoplasm of gum (C03*)
		C04: Malignant neoplasm of floor of mouth (C04*)
		C05: Malignant neoplasm of palate (C05.0)
		C06: Malignant neoplasm of other and unspecified parts of mouth (C06*)
	**oropharyngeal cancer**	C01: Malignant neoplasm of base of tongue (C01*)
		C02: Malignant neoplasm of other and unspecified parts of tongue (C02.4)
		C05: Malignant neoplasm of palate (C05.1, C05.2)
		C09: Malignant neoplasm of tonsil (C09*)
		C10: Malignant neoplasm of oropharynx (C10*)
	**unspecified oral and/or oropharyngeal cancers**	C02: Malignant neoplasm of other and unspecified parts of tongue (C02.8, C02.9)
		C05: Malignant neoplasm of palate (C05.8, C05.9)

*all subgroups included

#### Study population for epidemiological analysis

The prevalent population included patients with at least one hospital stay with selected ICD-10 codes as primary, related or associated diagnosis (Table 1). It was calculated for each year studied (*i*.*e*. 2011, 2012 and 2013). Patients with two or more ICD-10 codes belonging to different cancer locations were accounted for each of them.

The incident population of hospitalized patients with potentially HPV-related cancer(s) included patients with a first primary, related or associated diagnosis corresponding to one of the selected ICD-10 codes (Table 1), based on a longitudinal analysis for each year studied. Hospital stays were considered as the first hospital stay for potentially HPV-related cancers if there was no record of any selected ICD-10 code among diagnosis recorded within the previous 2-year period. Moreover, a second hospital stay with at least one ICD-10 code belonging to the same tumour anatomical site within a year after the first stay was required, to the exception of patients deceased within the first stay, who were included without this condition. As for prevalent population, those with two or more ICD-10 codes belonging to different cancer locations were accounted for each of them.

#### Study population for economic analysis

The economic analysis was performed on patients with a first in-hospital diagnosis of potentially HPV-related cancers in 2011. Patients who presented another primary tumour than those presented in Table 1 were excluded from the economic analysis. Patients with multiple cancer locations among potentially HPV-related cancers of interest were classified according to the location of their primary tumour.

### Collected data

The following information was extracted for each of selected patients: sex, age, number of hospital stays, and, for each stay, the corresponding DRG and the type of stay attributed through a stepwise backward classification for the following order: surgery, radiotherapy, chemotherapy, palliative care, other (*i*.*e*. stay with both surgery and chemotherapy was accounted as surgery). It is to be noticed that radiotherapy performed in ambulatory care and private hospitals are not recorded into the PMSI.

### Statistical analysis

#### Epidemiological analysis

Data are described using absolute and relative frequencies for categorical variables, and mean ± standard-deviation or median with interquartile ranges for quantitative variables, depending on their distribution.

Prevalence was calculated by dividing the number of prevalent patients by the total number of French women population for female genital cancers, or the total French population for other cancers, for the year corresponding to the studied year [22]. Incidence (*i*.*e*. number of new cases) was calculated the same way but dividing the number of incident hospitalised patients as defined in the “*Selection criteria and study populations*” section. Both results are presented by the total per 5-years age groups, and per gender where appropriate. Incidence rate was also calculated per 100,000 persons based on the French population size for each 5-years age groups [22].

A sensitivity analysis was conducted determining prevalence and incidence also including patients who presented cancers with location contiguous to uterus (ICD-10 code: C53.8), to vulva (C51.8) and to rectum, anus and anal canal (C21.8), as relation between HPV infection and those location contiguous cancers has not been formally proved to date.

Analyses were performed using SAS^®^ V9.3 software (SAS Institute Inc. Cary, NC, USA).

#### Economic analysis

In-hospital care costs were directly extracted from reimbursement tariffs completed in the PMSI database. In France, inpatient healthcare costs are reimbursed by the French National Health Insurance to hospitals through a national Diagnosis Related Group (DGR)-based payment system. The DRG of a hospital stay is notably determined based on recorded discharge diagnosis and classifying procedures performed during the stay. A reimbursement rate is defined for each DRG to cover treatments (except expensive drugs), medical procedures, nursing and physician fees. We used the published DRG tariffs for the year corresponding to the hospital stay. In-hospital care costs were described per patient, as mean ± standard-deviation (SD), and for the whole study population, per cancer location and per item of expenditure.

Since outpatient care costs are not available in the PMSI but represent a significant part of total direct medical costs related to cancer management, they were estimated based on the National Health Insurance Fund (*Caisse nationale de l'assurance maladie des travailleurs salaries*, Cnamts) report, which details expenditures by group of pathologies, treatments and health events, and daily allowances related to work interruption and disability pensions [23]. Outpatient care costs were estimated by applying distribution of expenditure for outpatient care active cancer from this report in regards of inpatient care costs calculated in our patients: 61.17% for inpatient expenditures, 33.83% for outpatient care, including 4.99% for disability allowances. They were estimated for the whole study population, per cancer location.

Costs are reported in Euros (€), year 2018, with costs prior to 2018 being revalued according to a consumer price index published by INSEE [22]. Healthcare costs were estimated by type of stay (surgery, radiotherapy, chemotherapy, palliative care, other).

### Complementary analysis based on fraction of cancer attributable to HPV

Based on the PAFs for the number of cancers attributable to HPV, we estimated as part of a complementary analysis (i) prevalence and incidence of patients that would be HPV positive among those identified for the different cancer locations, and (ii) the amount of health care costs that would actually be due HPV-related cancers for each of the cancer locations. From a literature review, Shield *et al*. study has been identified as the most suitable source of data, since it provides the most recent PAF values obtained in France, for all cancer locations assessed in the present study [20]. It should be noticed that PAF value was aggregated for vulva and vaginal cancers, which lead to the conduct of the complementary analysis on this gathered group. In addition, ICD-10 codes used to identify oral and oropharyngeal cancers in Shield *et al*. study (C02-06 for oral cancers, C01 and C09-10 for oropharyngeal cancers) were less finely distributed between oral and oropharyngeal cancers compared to our study (Table 1). We thus performed the complementary analysis using the PAF value for the whole group of HNCs.

Finally, PAF value used to perform the complementary analysis were 100.0% for cervical cancers, 23.0% for the gathered group of vulva and vagina cancers, 91.3% for anal cancer, and 18.5% for the whole group of HNCs.

### Ethics

Processing and exploitation of the data was done with respect to the Chair of the French data protection authority (*Commission Nationale de l'Informatique et des Libertés*, CNIL), including confidentiality at the individual level. Indeed, **stève** consultants, consultancy company responsible for the study analysis design and implementation, is approved by the CNIL for confidentiality, expertise and independence commitment criteria for access to French Health Data (http://doc.indsante.fr/20171128-%20Engagement%20de%20conformite%20au%20%20r%C3%A9f%C3%A9rentiel.pdf). Its CNIL authorization number for permanent access to PMSI data is n°1976885.

## Results

### Epidemiological results

Prevalence and incidence (*i*.*e*. number of new cases) are presented for the year 2013; data for the years 2011 and 2012, which were very similar to 2013, are available in supplementary tables (S1 Table).

#### Female genital cancers

A total of 7,597 women were hospitalized for cervical cancer, 1,491 for vulvar and 748 for vaginal cancer. The number of new cases of hospitalized women was 3,120, 522 and 323 for cervical, vulvar and vaginal cancers, respectively, corresponding to 9.23, 1.54 and 0.96 cases per 100,000 women (Table 2).

**Table 2:** Incident rate of potentially HPV-related female genital cancers among hospitalised women, per 100,000 French women, and of potentially HPV-related anal and head and neck cancers among hospitalised patients, per 100,000 French persons, by 5-years age groups, in 2013 in France.

	Female genital cancer			Anal cancer	Head and Neck cancers (HNCs)		
Age group	Cervical cancer	Vulvar cancer	Vaginal cancer	-	Oral cancer	Oropharyngeal cancer	Oral and/or unspecified oropharyngeal cancer
**≥100**	0.00	16.81	5.60	4.79	9.58	9.58	9.58
**95–99**	3.93	5.24	3.93	5.29	10.57	3.17	1.06
**90–94**	14.87	9.46	2.70	6.88	11.01	3.73	1.77
**85–89**	13.82	10.46	3.61	7.19	12.03	9.27	2.84
**80–84**	15.35	7.59	3.49	6.92	12.71	13.46	2.57
**75–79**	15.72	4.77	3.75	7.00	13.33	18.53	2.24
**70–74**	16.82	3.89	2.96	7.76	13.12	24.55	1.81
**65–69**	16.34	2.83	2.24	7.61	14.94	32.51	3.37
**60–64**	15.63	1.92	1.92	5.75	14.93	34.47	2.60
**55–59**	16.55	1.39	1.34	4.83	13.96	31.90	2.82
**50–54**	16.13	0.94	0.67	4.05	11.03	23.02	1.60
**45–49**	16.57	1.21	0.74	2.93	4.66	8.84	0.86
**40–44**	14.96	0.74	0.39	1.05	2.04	3.11	0.35
**35–39**	8.43	0.24	0.05	0.41	0.70	1.03	0.22
**30–34**	4.16	0.29	0.05	0.07	0.66	0.29	0.07
**25–29**	1.71	0.05	0.00	0.05	0.41	0.15	0.08
**<25**	0.31	0.00	0.00	0.00	0.30	0.10	0.08
**Overall**	**9.23**	**1.54**	**0.96**	**2.53**	**5.52**	**10.38**	**1.01**

**Fig 1:**
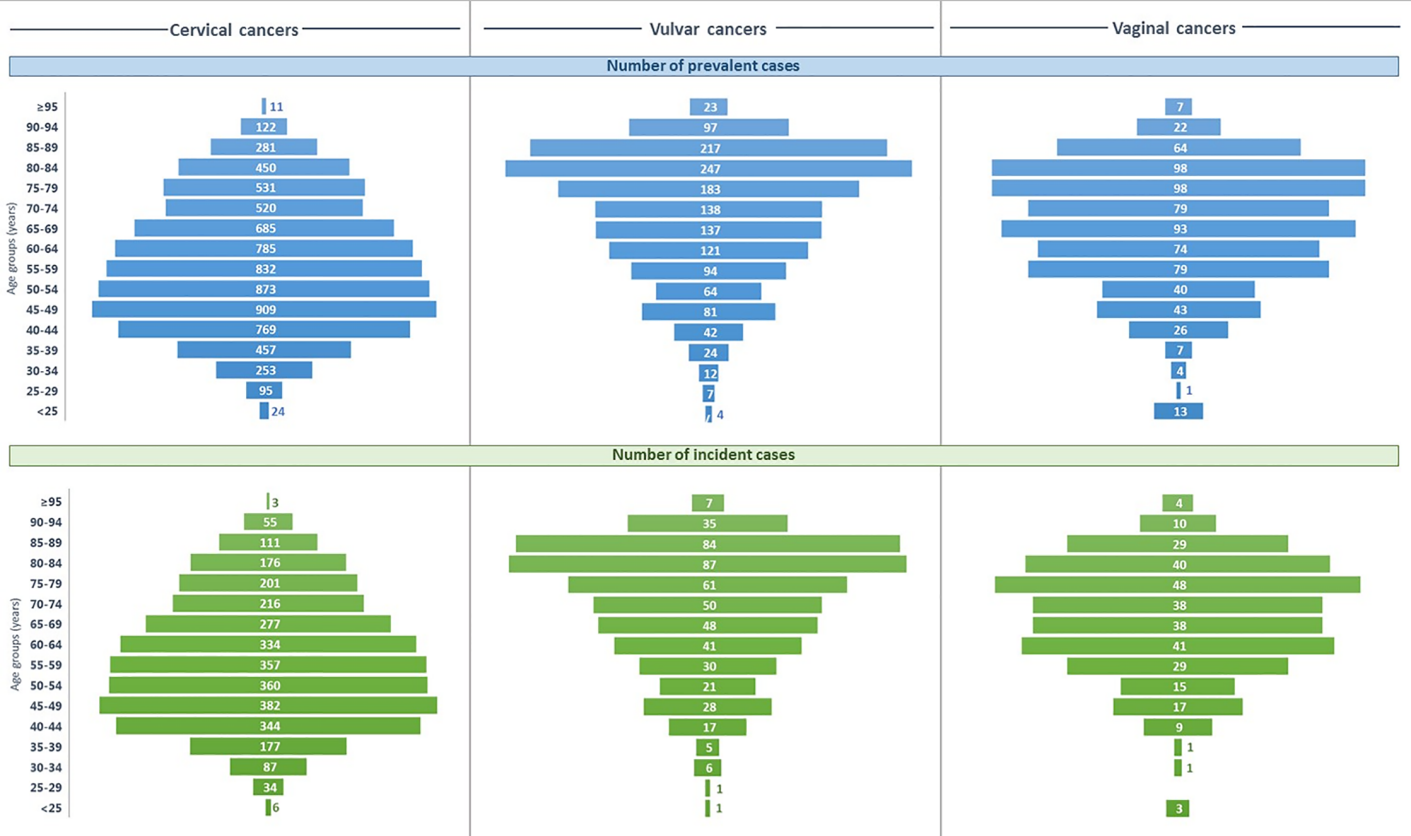
Pyramids of prevalent and incident cases of potentially HPV-related female genital cancers in 2013 in France. Population pyramids report the number of prevalent cases (upper side) and new cases (lower side) of potentially HPV-related female genital cancers, including cervical, vulvar and vaginal cancers, among hospitalised women, by 5-years age groups, in 2013 in France.

For cervical cancers, highest prevalence and incidence–both number of new cases and incidence rate—were observed in the 40–49 years’ age group; high prevalence and number of new cases persisted in adjacent older age groups prior to progressively decrease (Fig 1). Incidence rate showed a stable rate from the 40–49 years’ age group (14.96 per 100,000) to the 90–94 years’ age group (14.87 per 100,000; Table 2). For vulvar cancers, prevalence and number of new cases steadily increased from younger age groups to reach a maximum in 80–84 years’ age group, prior to decrease for very old age groups (Fig 1); related to the distribution of the general population age, incidence rates reached highest values in the 85–89 years’ age group (10.46 per 100,000; Table 2). For vaginal cancers, prevalence and number of new cases remain high and stable from 70–74 years old to 85–89 years old, prior to decrease for very old age groups (Fig 1); incidence rates reached highest values from the 70–74 years’ age group (3.75 per 100,000), increasing to a maximum of the oldest age groups (Table 2).

The sensitivity analysis including tumours contiguous to uterus and vulva showed similar results concerning the 5-years age-group distribution, with a prevalent population of 8,259 and 1,676 women hospitalized in 2013 for cervical and vulvar cancers, respectively, corresponding to 3,584 and 602 new cases or an incident rate of 10.60 and 1.78 per 100,000 persons.

Lastly, the complementary analysis estimated at 7,597 the number of women hospitalized for cervical cancer attributable to HPV in 2013, and 515 for both vulvar and vaginal cancers attributable to HPV. Similarly, the number of new cases of hospitalized women with cervical cancer attributable to HPV was estimated at 3,120, and at 194 for both vulvar and vaginal cancers.

#### Anal cancers

**Fig 2:**
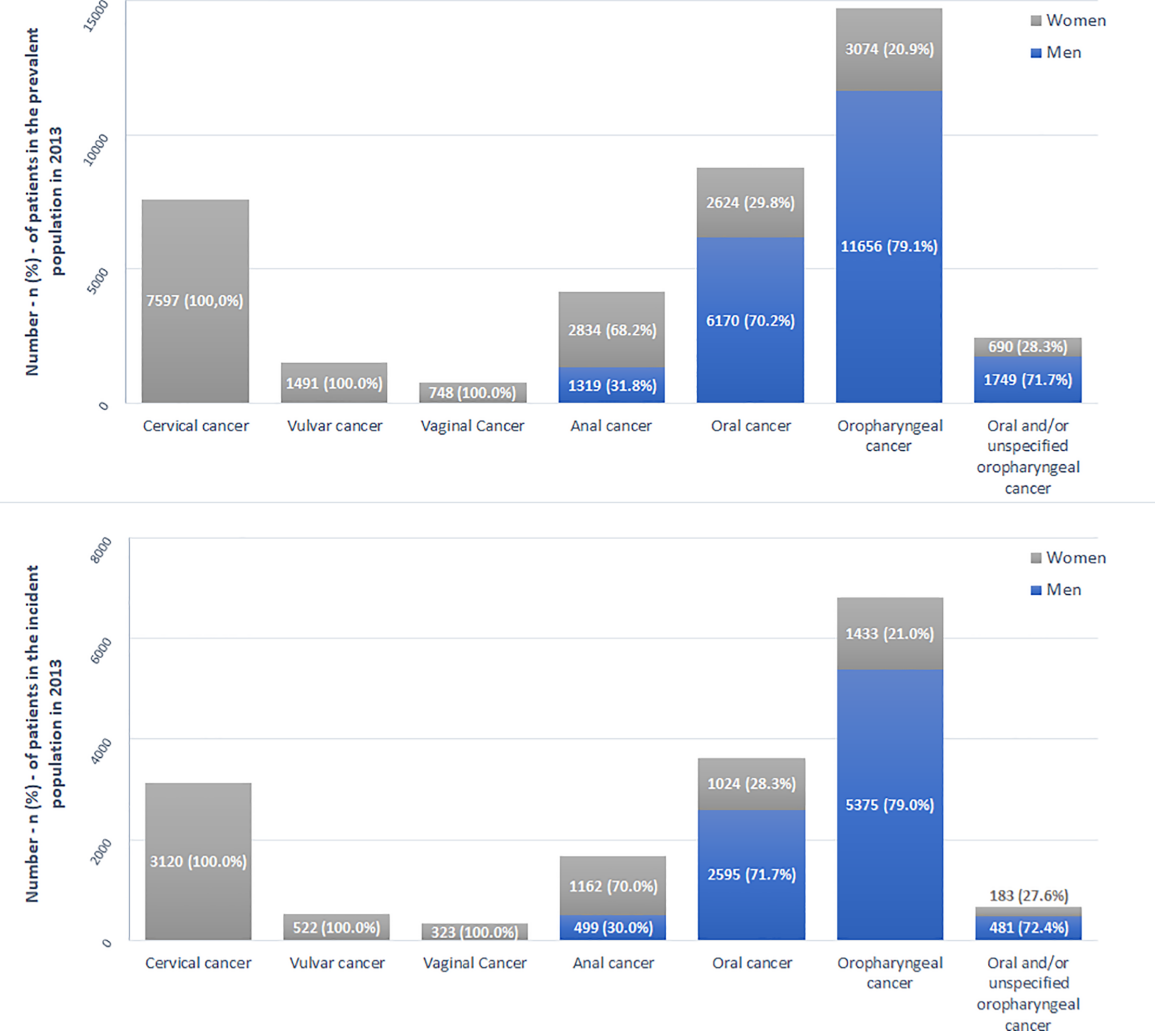
Repartition of men and women among prevalent and incident cases of potentially HPV-related cancers in 2013 in France. Repartition of men and women among prevalent cases (upper side) and new cases (lower side) are presented for patients hospitalized in 2013 with female genital cancers—including cervical, vulvar and vaginal cancers -, anal and head and neck cancers—including oral, oropharyngeal and oral and/or unspecified oropharyngeal cancers.

**Fig 3:**
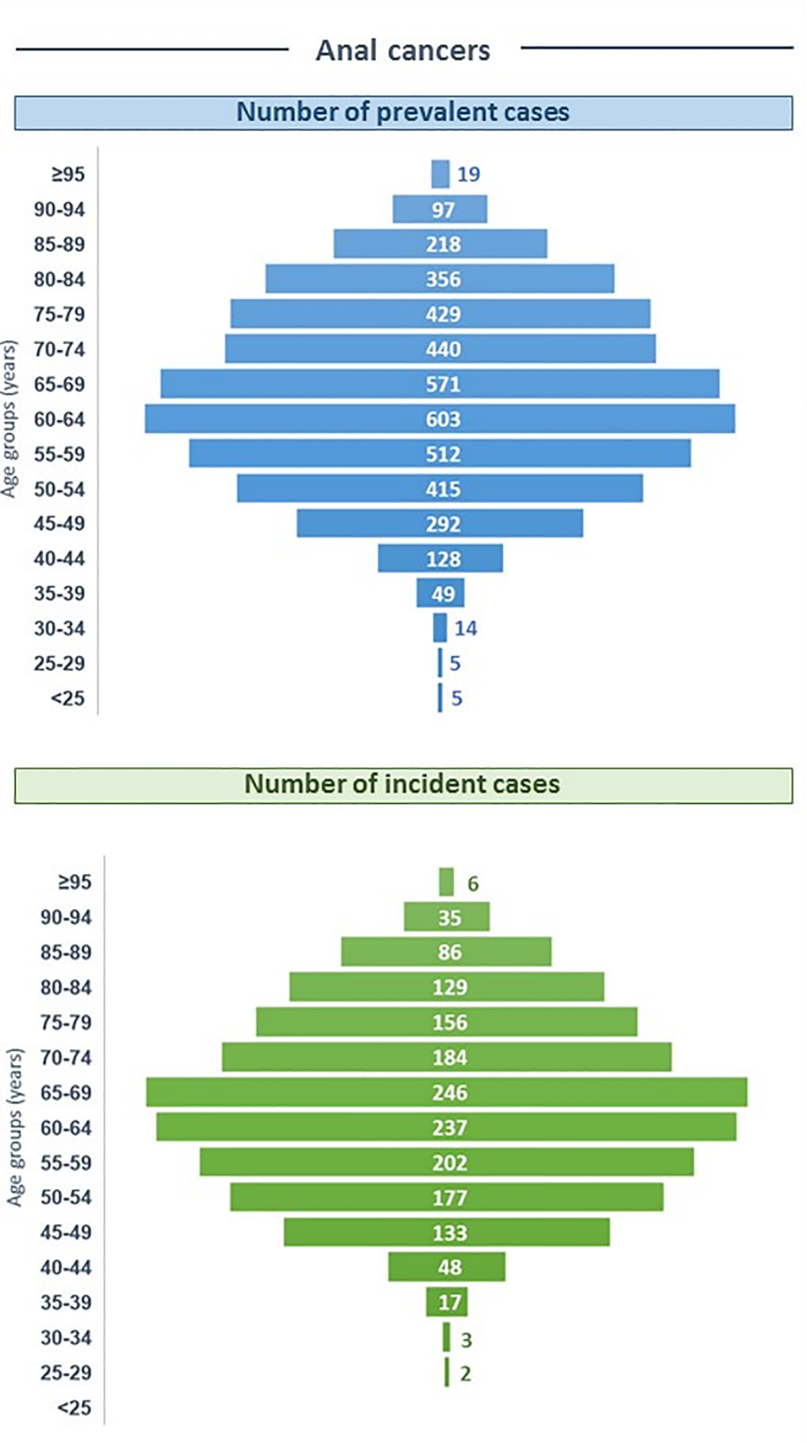
Pyramids of prevalent and incident cases of potentially HPV-related anal cancers in 2013 in France. Population pyramids report the number of prevalent cases (upper side) and new cases (lower side) of potentially HPV-related anal cancers among hospitalised patients, by 5-years age groups, in 2013 in France.

A total of 4,153 patients were hospitalized in 2013 for anal cancer, with a mean age of 65.4±13.2 years old; most of patients were women (68.2%; Fig 2). Overall, 1,661 new cases were observed (women 70.0%); this corresponded to an annual incidence of 2.53 per 100,000 persons (Table 2). The mean age of new cases was 65.0±12.9 years old. A constant increase in prevalence and number of new cases was observed to reach highest values in the 60–69 years’ age group prior to slightly decrease in older age groups (Fig 3). The incidence rates increased from younger age groups to reach highest values in the 65–69 and 70–74 years’ age group (7,61 and 7.76 per 100,000 persons, respectively; Table 2).

The sensitivity analysis including tumours contiguous to rectum, anus and anal canal, led to 5,139 patients hospitalized and 2,148 new cases in 2013, with similar age distribution, corresponding to an incident rate of 3.28 per 100,000 persons.

From the complementary analysis, the number of women hospitalized for anal cancer attributable to HPV was estimated at 3,792, and the number for new cases at 1,516.

#### Head and Neck cancers (HNCs)

**Fig 4:**
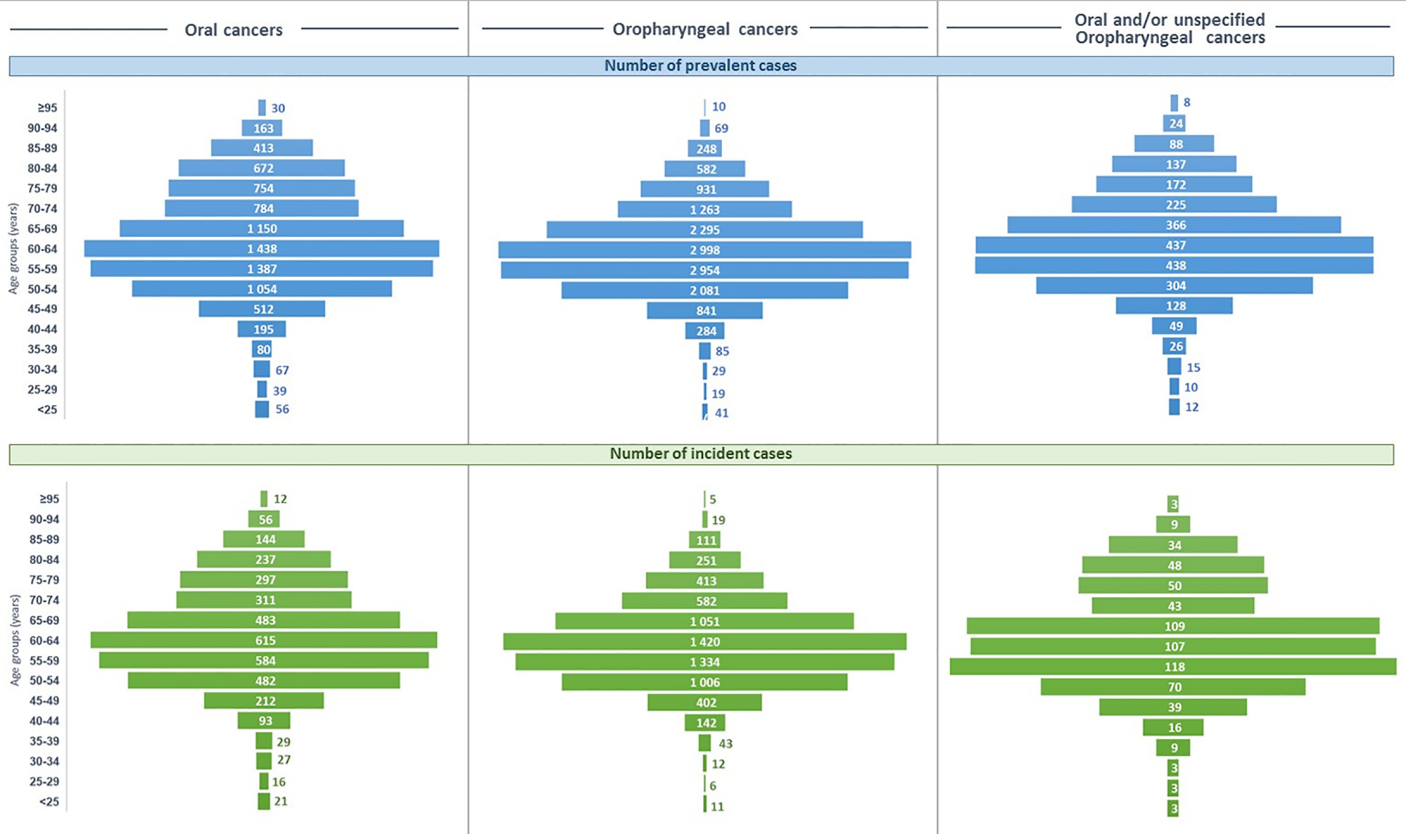
Pyramids of prevalent and incident cases of potentially HPV-related head and neck cancers in 2013 in France. Population pyramids report the number of prevalent cases (upper side) and new cases (lower side) of potentially HPV-related head and neck cancers (HNC), including oral, oropharyngeal and oral and/or unspecified oropharyngeal cancers, among hospitalised patients by 5-years age groups, in 2013 in France.

A total of 8,794 (men 70.2%) and 14,730 (men 79.1%) patients were hospitalized for oral and oropharyngeal cancer, respectively, with a mean age of 64.0±13.3 and 61.9±10.4 years old; 2,439 patients (men 71.7%) were hospitalized for oral and/or unspecified oropharyngeal cancer, with a mean age of 63.0±12.1 years old (Fig 2). Overall, annual incidence was 5.51 (3,619 new cases; men 71.7%), 10.38 (6,808 new cases; men 79.0%) and 1.01 (664 new cases; men 72.4%) per 100,000 persons for cancer of the oral cavity, oropharyngeal cancer, and oral and/or unspecified oropharyngeal cancer respectively (Table 2); their mean age was 63.3±12.9, 61.7±10.2 and 63.7±12.9 years old, respectively. For all HNC locations, prevalence and number of new cases increased to reach a maximum in 50–69 years’ age groups and decreased thereafter (Fig 4). The distribution of the incidence rates for oral and oropharyngeal cancers progressively increased from younger age groups to reaching its highest values from the 60–64 years’ age group (14.93 and 34.47 per 100,000, respectively for oral and oropharyngeal; Table 2). Five-years age group incidence rates for oral and/or unspecified oropharyngeal cancers remain relatively stable from the 50–54 years’ age group (1.6 per 100,000) to the oldest age groups (Table 2).

The complementary analysis allowed estimating at 4,803 the number of women hospitalized for HNC attributable to HPV, and at 2,052 the number for new cases.

### Economic analysis

The total number of patients accounted for the economic analysis—*i*.*e*. with a first hospital admission for potentially HPV-related cancer of interest in 2011, and after exclusion of patients with another primary tumour—was 14,388 patients. Among female genital cancer, the number of women was 3,005 for cervical cancer, 471 for vulvar cancer, and 279 for vaginal cancer; their mean age was 57.7±15.4, 71.7±14.3 and 66.5±16.6 years old for cervical, vulvar and vaginal cancer, respectively. The number of patients for anal cancer was 1,476, with a mean age of 65.2±13.4 years old. Similarly, the number of patient with potentially HPV-related oral cancer accounted in the economic analysis was 3,139 for oral cancers, and 6,018 for oropharyngeal cancers, with a mean age of 62.6±12.7 and 61.0±10.2 years old, respectively.

Patients with cervical cancers equally benefited from surgery (n = 1,630, 54.2%), chemotherapy (n = 1,769, 58.9%) and radiotherapy (n = 1,742, 58.0%; Table 3). Most of patients with vulvar and oral cancers were treated with surgery procedures (n = 332, 70.5% and n = 1,967, 62.7%, respectively). Oropharyngeal cancers were primarily treated with chemotherapy (n = 3,933, 65.4%). Patients with vaginal cancer mainly benefited from radiotherapy (n = 176, 63.1%). Lastly, anal cancers were mainly treated using both chemotherapy (n = 956, 64.8%) and radiotherapy (n = 772, 52.3%).

**Table 3:** Distribution of the type of hospital stay for patients accounted for the economic analysis, *i*.*e*. with a first hospital admission for potentially HPV-related cancer of interest in 2011, to the exclusion of patients with another primary tumour.

	Female genital cancer			Anal cancer	Head and Neck cancers (HNCs)	
	Cervical cancer	Vulvar cancer	Vaginal cancer	-	Oral cavity cancer	Oropharyngeal cancer
Surgery	1 630 (54.2%)	332 (70.5%)	82 (29.4%)	455 (30.8%)	1 967 (62.7%)	1 937 (32.2%)
Chemotherapy	1 769 (58.9%)	127 (27.0%)	123 (44.1%)	956 (64.8%)	1 371 (43.7%)	3 933 (65.4%)
Inpatient radiotherapy	1 742 (58.0%)	158 (33.5%)	176 (63.1%)	772 (52.3%)	1 267 (40.4%)	2 673 (44.4%)
Palliative care	745 (24.8%)	151 (32.1%)	77 (27.6%)	263 (17.8%)	804 (25.6%)	1 546 (25.7%)

**Fig 5:**
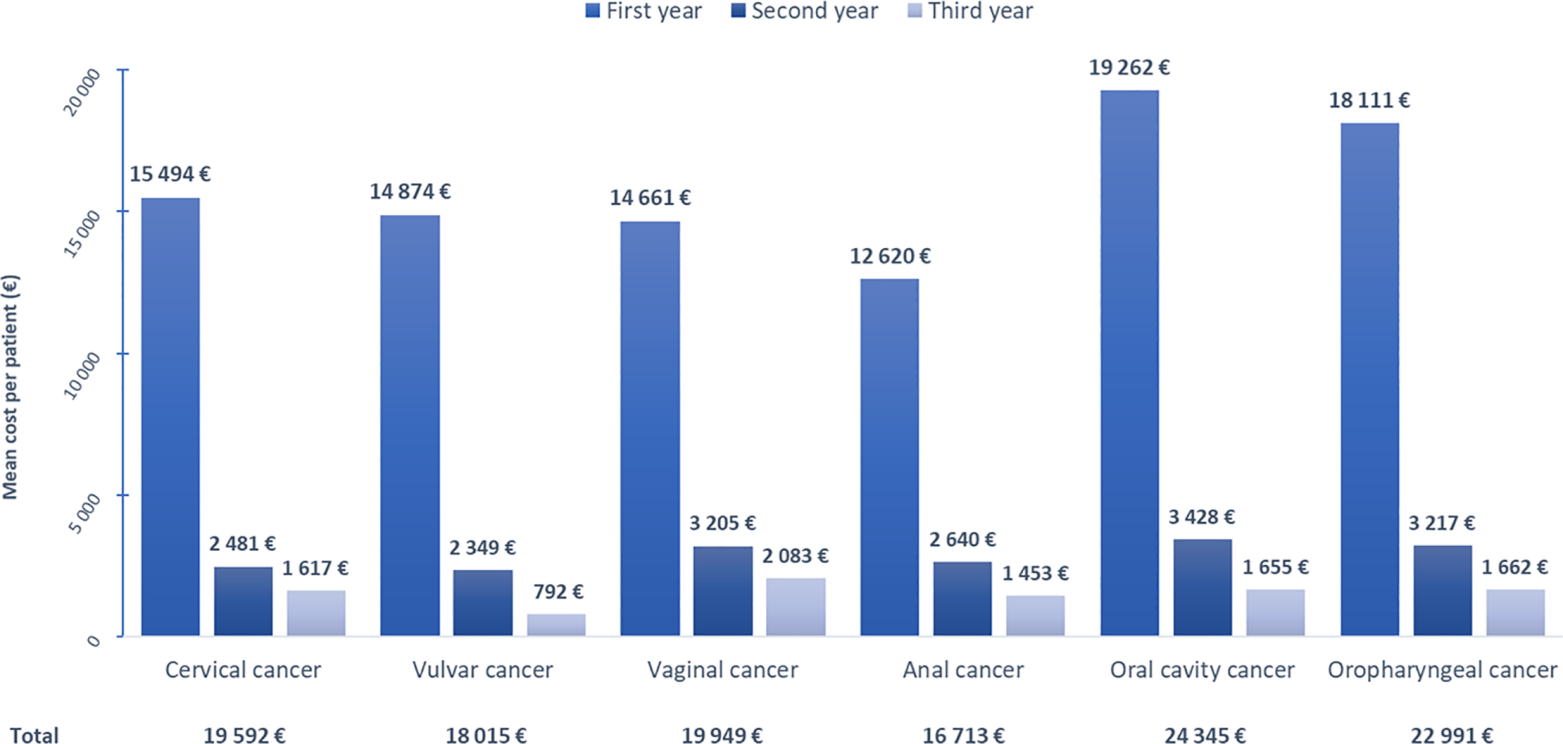
Mean hospital care costs per patient and per year over the 3-year period after in-hospital potentially HPV-related cancer diagnosis. Mean hospital care costs distribution is presented per patient accounted for the economic analysis - *i*.*e*. with a first hospital admission for potentially HPV-related cancer of interest in 2011, to the exclusion of patients with another primary tumour—over the 3 years after cancer diagnosis.

Within 3 years after potentially HPV-related cancer in-hospital diagnosis, the average total direct medical cost of hospital care per patient for potentially HPV-related cancers were comparable, varying from 17,047 € for anal cancer to 24,832 € for oral cancer (Fig 5; Table 4). Since most hospital admission occurred during the first year of care, hospital care costs were largely concentrated in the first years after in-hospital diagnosis (Fig 5). Relatively, very low hospital care costs were recorded during the second and third years after diagnosis.

**Table 4:** In-hospital medical care cost distribution per patient and cancer location for patients—*i*.*e*. with a first hospital admission for potentially HPV-related cancer of interest in 2011, to the exclusion of patients with another primary tumour—within the 3 years after cancer diagnosis, extracted from the PMSI (reimbursement tariffs).

Item of in-hospital direct medical costs	Cervical cancer	Vulvar cancer	Vaginal cancer	Anal cancer	Oral cancer	Oropharyngeal cancer
**Surgery (€, per patient)** mean (SD)	4,775 (6,093)	6,302 (6,892)	2,971 (6,478)	3,149 (7,062)	8,173 (9,698)	3,553 (7,317)
**Chemotherapy (€, per patient)** mean (SD)	4,677 (8,895)	2,706 (6,915)	4,774 (8,914)	4,195 (8,096)	5,260 (11,557)	7,289 (12,037)
**Radiotherapy (€, per patient)** mean (SD)	2,988 (3,736)	1,711 (3,176)	3,305 (4,162)	2,822 (3,638)	2,177 (3,619)	2,503 (3,866)
**Palliative care (€, per patient)** mean (SD)	2,255 (5,857)	3,143 (6,171)	2,848 (5,944)	1,605 (4,813)	2,409 (5,818)	2,396 (5,701)
**Other hospitalizations (€, per patient)** mean (SD)	5,290 (8,368)	4,514 (6,687)	6,450 (8,002)	5,276 (7,278)	6,814 (9,457)	7,710 (9,725)
**Total in-hospital costs (€, per patient)** **mean (SD)**	**19,984 (17,800)**	**18,376 (14,593)**	**20,348 (19,282)**	**17,047 (16,533)**	**24,832 (21,443)**	**23,450 (20,317)**

**Fig 6:**
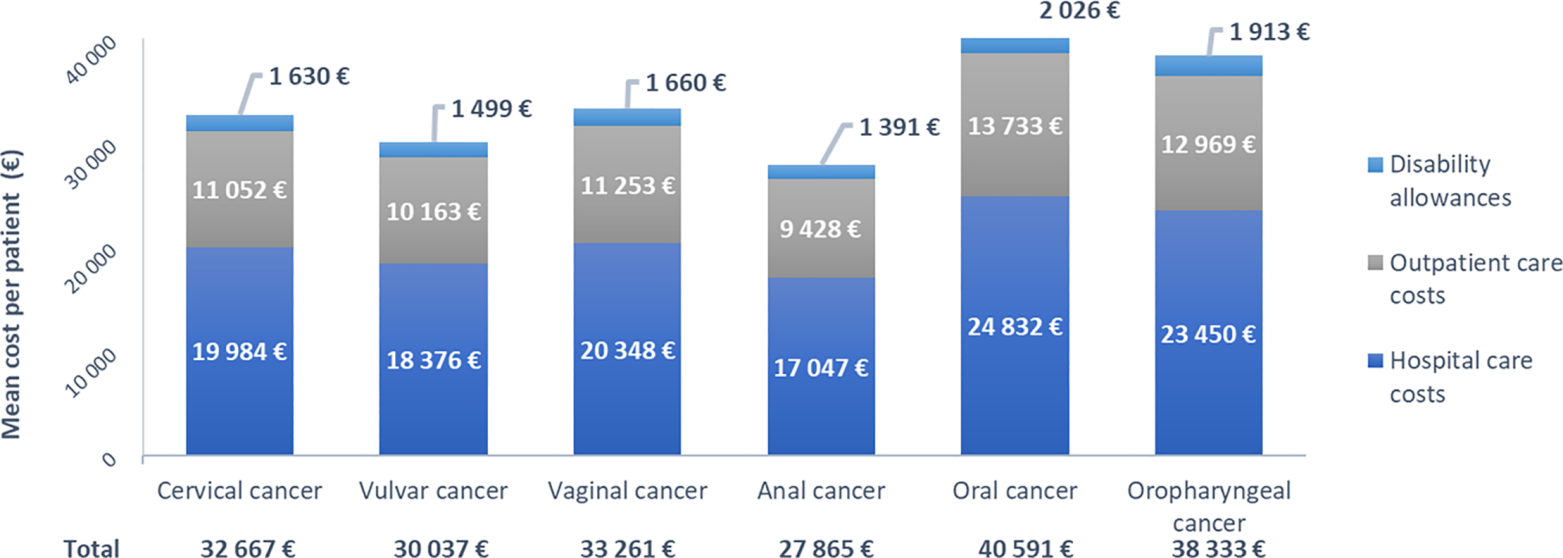
Mean hospital and ambulatory care costs per patient over the 3-year period after in-hospital potentially HPV-related cancer diagnosis. Mean care costs, including including hospital costs, primary care, daily allowances and disability pensions, are presented per patient accounted for the economic analysis—*i*.*e*. with a first hospital admission for potentially HPV-related cancer of interest in 2011, to the exclusion of patients with another primary tumour—over the 3 years after cancer diagnosis.

Based on the Cnamts report on distribution of expenditure for outpatient care active cancer [23], estimation of total cost related to potentially HPV-related cancers within the 3 years after in-hospital diagnosis, including hospital costs, primary care, daily allowances and disability pensions ranged from about €31 K for female genital and anal cancers (32,666 €, 30,037 €, and 33,262 €, respectively for cervical, vulvar and vaginal cancers, and 27,865 € for anal cancer) to about €41 K for HNCs (40,591 € and 38,333 €, respectively for oral and oropharyngeal cancers; Fig 6, Table 4).

Considering the total population with a first hospital admission for potentially HPV-related cancer in 2011, total costs associated to HPV-related cancers were estimated to nearly €511 M, divided into 1/3 for female genital and anal cancers (€103 M, €14 M, and €8 M respectively for cervical, vulvar and vaginal cancers, and €42 M for anal cancer; Table 5), and 2/3 for HNCs (€121 M for oral cancer and €222 M for oropharyngeal cancer; Table 5). Distribution of expenditure among the different therapeutic management largely varied depending on the cancer location. According to therapeutic strategies mostly used in the different cancers, hospital care costs for vulvar and anal cancers were mainly related to chemotherapy, while they were rather attributed to surgery for vulvar cancer. From the complementary analysis, amounts of health care costs for cancer attributable to HPV were estimated to reach €103 M and €5 M respectively for cervical and both vulvar and vaginal cancers, €38 M for anal cancer, and €63 M for HNCs.

**Table 5:** Total cost distribution per cancer location for patients accounted for the economic analysis—i.e. with a first hospital admission for potentially HPV-related cancer of interest in 2011, to the exclusion of patients with another primary tumour—within the 3 years after cancer diagnosis.

	Cervical cancer	Vulvar cancer	Vaginal cancer	Anal cancer	Oral cancer	Oropharyngeal cancer
**Hospitalization** (€, total)	63,176,657	8,530,148	4,958,878	25,608,046	74,162,931	135,806,176
*Surgery*	*14*,*547*,*253*	*2*,*905*,*665*	*709*,*212*	*4*,*726*,*047*	*22*,*868*,*744*	*19*,*306*,*984*
*Chemotherapy*	*15*,*934*,*772*	*1*,*281*,*989*	*1*,*152*,*349*	*6*,*657*,*740*	*16*,*882*,*862*	*44*,*656*,*453*
*Radiotherapy*	*8*,*423*,*416*	*781*,*925*	*844*,*588*	*4*,*006*,*579*	*6*,*257*,*942*	*13*,*642*,*448*
*Palliative care*	*7*,*282*,*808*	*1*,*435*,*552*	*701*,*102*	*2*,*368*,*929*	*7*,*300*,*735*	*13*,*712*,*741*
*Other hospitalizations*	*16*,*988*,*406*	*2*,*125*,*017*	*1*,*551*,*627*	*7*,*848*,*754*	*20*,*852*,*647*	*44*,*487*,*551*
**Outpatient care costs**	34,939,779	4,717,590	2,742,502	14,162,501	41,015,726	75,107,454
**Disability allowances**	5,153,695	695,855	404,525	2,089,001	6,049,910	11,078,516
**Total costs**	**103,270,131**	**13,943,593**	**8,105,905**	**41,859,548**	**121,228,567**	**221,992,147**

## Discussion

The objectives of this study were to estimate prevalence and number of new cases of potentially HPV-related cancers based on in-hospital diagnosis information, and to evaluate costs related to their management. We used the national database of French public and private hospital information (*Programme de Médicalisation des Systèmes d’Information*, PMSI), permitting the study of potentially HPV-related cancer epidemiology and costs, including rare locations such as anus, vulva and vaginal.

### Main findings

#### Female genital cancers

According to the physiopathological process of cervical malignant tumour development, almost all cervical cancers are expected to be related to HPV (1 to 2% of cervical cancers negative for HPV—such as gastric-type or mesonephric adenocarcinomas–are considered as biologically distinct subset of tumours [24–26]). The National Cancer Institute (INCa) estimated in 2012 a total of 3,028 new cervical cancer cases in France [27], which is very close to the 3,120 incident patients hospitalized for cervical cancer in 2013 in our study. Recently, INCa projected 2,797 new cases of cervical cancer in 2015 [28], confirming the decline in the number of cases observed during the last decade. This decline was also observed in our study, with 3,221, 3,201 and 3,120 newly hospitalized women in 2011, 2012 and 2013 for cervical cancer, respectively, corresponding to a 3.1% decrease.

To our knowledge, no French data for vulvar and vaginal HPV potentially-related cancers are available [7,12]. In Europe, the prevalence of HPV in vulvar and vaginal cancers has been estimated at 19.3% and 71.1%, respectively [3]. Due to the low number of vulvar and vaginal cancers, their incidence is difficult to estimate using French registries. A French study conducted in 1997 estimated the number of new cases for vulvar and vaginal cancers to be 0.9 and 1.7 per 100,000, respectively [29]. A more recent one estimated between 600 and 700 cases in 2000, respectively for both cancers [30]. Lastly, Borget *et al*. reported a total number of 1,237 patients hospitalized for vulvar cancer and 728 for vaginal cancer in 2006 [31]. We identified 522 new cases for vulvar cancer and 323 new cases for patient with new in-hospital diagnosis for vaginal cancer in 2013. We estimated the incidence rate at 1.54 cases per 100,000 women for vulvar cancers, and 0.96 for vaginal cancers, confirming this increase in the number of new cases for vulvar cancer in France. Accordingly to our results for France, a study based on Dutch registries also reported a steady increase in the number of new cases for vulvar cancers between 1989 and 2010, from 2.0 to 2.7 new cases per 100,000 women (standardized Europe) [32].

#### Anal cancers

A French study reported that anal cancer has been attributed to HPV in about 96% of cases, more particularly to HPV 16/18 in 78% of cases [9]. In our study, we reported 2.5 new cases per 100,000 in 2013. Between 1992 and 2005, FRANCIM cancer registry estimated the annual number of new cases for anal cancer at 1.4 new cases per 100,000 persons [33]. Concerning prevalent cases, Abramowitz *et al*. estimated the size of the prevalent population of patients hospitalized for anal cancer in 2006 from the PMSI database at 3,640 patients [34]. Compared to the 4,153 patients hospitalized in 2013 identified in our study, this would correspond to an increase of 14.2%. Similar increase in the number of cases of anal cancer are reported in other European countries. UK reports an increase of more than 130% since the 1970s, especially among women (+ 191%), with an incidence rate of anal cancer (standardized Europe) in 2013 of 2.1 cases per 100,000 people [35]. Similarly, a study of the Danish registries reported an increase of anal cancer incidence between 1978–1982 and 2003–2008 of 117% in women and 78% in men [36].

#### Head and Neck cancers (HNCs)

The prevalence of HPV in HNCs was found to be 46.5% and 10.5% for oropharyngeal and oral cancers in France, respectively, with HPV genotype 16 being responsible for 89.7% and 95.5% of them [10]. In our study, in 2013, 3,619 patients were newly hospitalized for oral cancer for an annual incidence of 5.52 per 100,000 people; 6,808 patients had a first hospital admission for oropharyngeal cancer, corresponding to an annual incidence of 10.38 per 100,000 persons. It is however difficult to compare these results with the existing literature since localization definitions vary between studies [37]. In addition, no epidemiological data on oral or oropharyngeal cancers has been identified in INCa reports [28].

#### Economic analysis

This study is the first to evaluate the direct medical cost of HPV potentially related cancers using an incident cohort over a period of 3 years follow-up.

We adopted a 3-year time horizon based on a published cost analysis from a Danish registry [38]. The assessment of health care costs associated to potentially HPV-related genital warts and cervical cancer for a 3-year period from cancer diagnosis showed that around two-thirds were concentrated on the first year. Health care costs over the second and third years were similar; they declined after the first year to approach costs recorded over the year preceding cancer diagnosis. In line with the French Health Authority guidelines for the choice of economic evaluation time horizon, we thus adopted a long enough time horizon to reflect all expected consequences in costs of potentially HPV-related cancers [39].

This economic analysis allowed an accurate calculation of hospital care costs and an estimation of totals cost for cancers care. Most expenditures were related to hospital care costs and were concentrated in the first year of care, which accounted for more the ¾ of total costs. Similar results were observed with the Danish registry that estimated health care costs of anal, penile, vaginal and vulvar cancer, with 2/3 of costs observed over the 3-year study period that were concentrated on the first year [38]. For hospital care, mean costs per patient for the 3-years follow-up ranged between €17 K and €20 K for female genital and anal cancers, and €23 K and €24 K for HNCs. Total care costs per patient varied between €30 K for female genital and anal cancers and €40 K for HNCs. Overall, considering the 14,388 patients with a first hospital admission for potentially HPV-related cancer of interest in 2011, after exclusion of patients with another primary tumour, the total economic burden associated with HPV-potentially related cancers was about €500 M for the French National Health Insurance during the 3 years after cancer diagnosis, divided into 1/3 for female genital and anal cancers (€167 M) and 2/3 for HNCs (€343 M). As observed in other European countries, most of health insurance expenditure associated to potentially HPV-related cancers concerned genital—mainly cervical—and oropharyngeal cancers [38,40]. Considering the amount of health care costs for cancer attributable to HPV based on PAF values from the literature, the total economic burden was about €200 M for the French National Health Insurance during the 3 years after cancer diagnosis.

### Strengths and limitations

The PMSI is a national comprehensive medico-administrative database that provides extensive data covering a population of more than 65 million French inhabitants [16,41]. The huge number of patients contained into this database and the almost comprehensive record of medical expenses have been shown to presents major advantages for epidemiological studies [16]. In addition, the linkage of prospectively collected individual health care consumption data through a unique anonymous identifier (*Numéro d’Identification au Repertoire*, NIR), allows performing both transversal and longitudinal analysis to accurately assess prevalence and incidence or new events at a nationwide level. Furthermore, comprehensiveness and very detailed levels of cost data are highly valuable to perform economic analysis.

This study has however to be interpreted in light of certain limitations due to the nature of the source of data. Indeed, errors in diagnosis coding would influence the epidemiological data; however, errors are expected to be very rare for these well-known diseases. In addition, quality control and audits are performed on PMSI data to identify aberrant or missing data [16,41].

A second limitation is the lack of detailed clinical data, which leads us to create a third group for HNCs gathering unspecified location cancers. In addition, due to the absence of biological results in the PMSI, we could not determine the effective part of HPV-related cancers among cases identified; however, we clearly stated that the study aimed at assessing epidemiology and burden of potentially HPV-related cancers. We still performed a complementary analysis to estimate the number of potentially HPV-related cancers identified through our study that would actually be related to HPV. We used PAF values available from the most recent French study in the literature [20]. However, we used aggregated PAF values for the group of vulva and vagina cancers as well as for HNCs due to the lack of data availability and transposability, respectively. Results have thus to be interpreted taking into account that, among vulva and vaginal cancers, vagina cancers are the rarest but associated with the highest PAF value (70% *versus* 15%) [21]. Among HNCs, oropharyngeal cancers are more common than oral cancers, and are associated to a higher PAF value (30% *versus* 4%) [20].

Finally, main limitations would concern the cost calculation. Indeed, only hospital care costs are recorded into the PMSI; ambulatory care costs and disability allowances were estimated extrapolating hospital care costs, applying distribution of care costs for active cancers reported by the Cnamts. The Cnamts examines the structure of expenditures by group of pathologies, treatments and health events in 2013 based on data from long-term disorders. However, the principle of full reimbursement of medical care related to long-term disorders may lead to a bias, when it includes abusively care unrelated to these specific diseases. This may result in slightly overestimating the part of expenditures related to primary care. On the other hand, the same distribution of expenses was used for all cancers, assuming that the work absenteeism and disability degrees were the same for all cancers in our study. Although this may be incorrect, in the absence of recent specific data, these extrapolations make it possible to estimate the most complete costs associated with HPV potentially related cancers. In addition, it is to be noticed that costs available in the PMSI do not include physician fees which are paid in addition to the DRG. Lastly, costs of radiotherapy are not available for private hospitals. Lastly, we had a conservative approach by including only patients with a single cancer in the economic analysis, which may lead to underestimate the total cost of health insurance expenditure.

## Conclusion

This study reported the most up-to-date epidemiological and cost data for potentially HPV-related cancers, including locations such as anus, vulva and vagina cancers, for which data were scarce. These cancers represent a significant cost to the Health Insurance. A substantial fraction of these cancer is related to HPV and most of them could be averted. These data may be used to evaluate the potential impact of new preventive strategies to reduce the number of new cases of these cancers, including the nine-valent vaccine, indicated in the prevention of cervical, vaginal, vulvar and anal cancers caused by specific HPV types.

## Supporting information

S1 Table Number of prevalent and incident cases of potentially HPV-related female genital, anal and head and neck cancers among hospitalised patients by 5-years age groups, in 2011 and 2012 in France. (DOCX)
