# Supplementary material for: Epidemiological and economic burden of potentially HPV-related cancers in France
Source: PLoS One. 2018 Sep 20;13(9):e0202564. doi: 10.1371/journal.pone.0202564 (PMC6147406; doi:10.1371/journal.pone.0202564)
Supplement: S1 Table — (DOCX) [file pone.0202564.s001.docx]

S1 Table – Number of prevalent and incident cases of potentially HPV-related female genital, anal and head and neck cancers among hospitalised patients by 5-years age groups, in 2011 and 2012 in France.

|  | Female genital cancer, n (%) | | | Anal cancer, n (%) | Head and Neck cancers (HNCs), n (%) | | |
| --- | --- | --- | --- | --- | --- | --- | --- |
| Age group | **Cervical cancer** | **Vulvar cancer** | **Vaginal cancer** | **-** | **Oral cancer** | **Oropharyngeal cancer** | **Oral and/or unspecified oropharyngeal cancer** |
| PREVALENT CASES | | | | | | | |
| *Year 2011* | | | | | | | |
| <25 ans | 32 (0,4%) | 5 (0,4%) | 14 (1,9%) | 7 (0,2%) | 44 (0,5%) | 20 (0,1%) | 4 (0,1%) |
| 25-29 | 99 (1,3%) | 6 (0,4%) | 2 (0,3%) | 7 (0,2%) | 33 (0,4%) | 16 (0,1%) | 8 (0,3%) |
| 30-34 | 238 (3,1%) | 8 (0,6%) | 4 (0,5%) | 18 (0,5%) | 47 (0,5%) | 30 (0,2%) | 16 (0,6%) |
| 35-39 | 523 (6,8%) | 16 (1,2%) | 14 (1,9%) | 49 (1,2%) | 103 (1,1%) | 86 (0,6%) | 28 (1,0%) |
| 40-44 | 775 (10,1%) | 46 (3,3%) | 25 (3,3%) | 140 (3,5%) | 224 (2,5%) | 326 (2,2%) | 73 (2,7%) |
| 45-49 | 905 (11,8%) | 67 (4,8%) | 37 (4,9%) | 261 (6,5%) | 602 (6,6%) | 1,046 (7,0%) | 168 (6,2%) |
| 50-54 | 878 (11,5%) | 85 (6,1%) | 46 (6,1%) | 394 (9,9%) | 1,131 (12,5%) | 2,297 (15,3%) | 354 (13,1%) |
| 55-59 | 802 (10,5%) | 92 (6,6%) | 76 (10,2%) | 502 (12,6%) | 1,558 (17,2%) | 3,188 (21,2%) | 536 (19,9%) |
| 60-64 | 784 (10,2%) | 102 (7,3%) | 77 (10,3%) | 592 (14,8%) | 1,527 (16,8%) | 2,957 (19,7%) | 481 (17,9%) |
| 65-69 | 607 (7,9%) | 122 (8,8%) | 84 (11,2%) | 488 (12,2%) | 991 (10,9%) | 1,929 (12,8%) | 327 (12,1%) |
| 70-74 | 559 (7,3%) | 141 (10,2%) | 91 (12,2%) | 420 (10,5%) | 786 (8,7%) | 1,286 (8,6%) | 208 (7,7%) |
| ≥75 | 1,449 (18,9%) | 699 (50,3%) | 278 (37,2%) | 1,122 (28,1%) | 2,022 (22,3%) | 1,848 (12,3%) | 490 (18,2%) |
| Total | **7,651** | **1,389** | **748** | **4,000** | **9,068** | **15,029** | **2,693** |
| *Year 2012* | | | | | | | |
| <25 ans | 33 (0,4%) | 9 (0,6%) | 14 (1,9%) | 8 (0,2%) | 49 (0,6%) | 31 (0,2%) | 11 (0,4%) |
| 25-29 | 117 (1,5%) | 6 (0,4%) | 3 (0,4%) | 6 (0,1%) | 35 (0,4%) | 19 (0,1%) | 7 (0,3%) |
| 30-34 | 258 (3,4%) | 16 (1,1%) | 5 (0,7%) | 17 (0,4%) | 56 (0,6%) | 28 (0,2%) | 17 (0,7%) |
| 35-39 | 476 (6,2%) | 20 (1,4%) | 13 (1,7%) | 59 (1,4%) | 94 (1,1%) | 77 (0,5%) | 28 (1,1%) |
| 40-44 | 797 (10,4%) | 41 (2,8%) | 26 (3,4%) | 124 (3,0%) | 217 (2,5%) | 294 (2,0%) | 55 (2,1%) |
| 45-49 | 906 (11,8%) | 82 (5,6%) | 33 (4,4%) | 269 (6,6%) | 546 (6,2%) | 976 (6,5%) | 168 (6,4%) |
| 50-54 | 908 (11,8%) | 81 (5,5%) | 54 (7,2%) | 404 (9,9%) | 1,124 (12,7%) | 2,225 (14,9%) | 336 (12,9%) |
| 55-59 | 789 (10,3%) | 86 (5,9%) | 72 (9,5%) | 488 (12,0%) | 1,439 (16,3%) | 3,081 (20,7%) | 464 (17,8%) |
| 60-64 | 809 (10,6%) | 103 (7,0%) | 84 (11,1%) | 605 (14,9%) | 1,464 (16,6%) | 2,987 (20,0%) | 475 (18,2%) |
| 65-69 | 643 (8,4%) | 127 (8,6%) | 86 (11,4%) | 504 (12,4%) | 1,063 (12,0%) | 2,123 (14,2%) | 372 (14,3%) |
| 70-74 | 514 (6,7%) | 154 (10,5%) | 84 (11,1%) | 404 (9,9%) | 789 (8,9%) | 1,242 (8,3%) | 228 (8,7%) |
| ≥75 | 1,417 (18,5%) | 745 (50,7%) | 280 (37,1%) | 1,184 (29,1%) | 1,961 (22,2%) | 1,823 (12,2%) | 448 (17,2%) |
| Total | **7,667** | **1,470** | **754** | **4,072** | **8,837** | **14,906** | **2,609** |
| INCIDENT CASES | | | | | | | |
| *Year 2011* | | | | | | | |
| <25 ans | 14 (0,4%) | 1 (0,2%) | 7 (2,1%) | 1 (0,1%) | 11 (0,3%) | 2 (0,0%) | 1 (0,1%) |
| 25-29 | 31 (1,0%) | 2 (0,4%) | 1 (0,3%) | 1 (0,1%) | 12 (0,3%) | 5 (0,1%) | 2 (0,3%) |
| 30-34 | 93 (2,9%) | 1 (0,2%) | 3 (0,9%) | 3 (0,2%) | 20 (0,5%) | 9 (0,1%) | 2 (0,3%) |
| 35-39 | 207 (6,4%) | 6 (1,2%) | 8 (2,4%) | 19 (1,2%) | 43 (1,2%) | 38 (0,5%) | 5 (0,7%) |
| 40-44 | 329 (10,2%) | 10 (2,0%) | 9 (2,7%) | 58 (3,7%) | 113 (3,1%) | 167 (2,4%) | 25 (3,4%) |
| 45-49 | 388 (12,0%) | 27 (5,3%) | 13 (4,0%) | 116 (7,3%) | 254 (6,9%) | 521 (7,5%) | 40 (5,4%) |
| 50-54 | 389 (12,1%) | 24 (4,7%) | 21 (6,4%) | 158 (10,0%) | 502 (13,7%) | 1,069 (15,5%) | 99 (13,5%) |
| 55-59 | 349 (10,8%) | 42 (8,3%) | 36 (10,9%) | 206 (13,0%) | 661 (18,1%) | 1,475 (21,3%) | 129 (17,5%) |
| 60-64 | 323 (10,0%) | 35 (6,9%) | 35 (10,6%) | 238 (15,0%) | 639 (17,5%) | 1,422 (20,6%) | 127 (17,3%) |
| 65-69 | 291 (9,0%) | 46 (9,1%) | 36 (10,9%) | 189 (11,9%) | 380 (10,4%) | 858 (12,4%) | 102 (13,9%) |
| 70-74 | 229 (7,1%) | 63 (12,4%) | 44 (13,4%) | 163 (10,3%) | 308 (8,4%) | 541 (7,8%) | 61 (8,3%) |
| ≥75 | 578 (17,9%) | 250 (49,3%) | 116 (35,3%) | 431 (27,2%) | 717 (19,6%) | 810 (11,7%) | 143 (19,4%) |
| Total | **3,221** | **507** | **329** | **1,583** | **3,660** | **6,917** | **736** |
| *Year 2012* | | | | | | | |
| <25 ans | 6 (0,2%) | 2 (0,4%) | 3 (1,0%) | 4 (0,2%) | 18 (0,5%) | 3 (0,0%) | 7 (1,0%) |
| 25-29 | 35 (1,1%) | 1 (0,2%) | 1 (0,3%) | 0 (0,0%) | 17 (0,5%) | 9 (0,1%) | 1 (0,1%) |
| 30-34 | 86 (2,7%) | 4 (0,8%) | 2 (0,7%) | 9 (0,6%) | 21 (0,6%) | 13 (0,2%) | 5 (0,7%) |
| 35-39 | 211 (6,6%) | 10 (2,1%) | 1 (0,3%) | 26 (1,6%) | 39 (1,1%) | 37 (0,5%) | 7 (1,0%) |
| 40-44 | 347 (10,8%) | 7 (1,5%) | 13 (4,4%) | 54 (3,3%) | 89 (2,5%) | 150 (2,2%) | 15 (2,1%) |
| 45-49 | 362 (11,3%) | 21 (4,4%) | 17 (5,8%) | 102 (6,3%) | 239 (6,8%) | 486 (7,1%) | 49 (7,0%) |
| 50-54 | 375 (11,7%) | 24 (5,0%) | 18 (6,1%) | 165 (10,2%) | 497 (14,1%) | 1,066 (15,6%) | 89 (12,7%) |
| 55-59 | 365 (11,4%) | 34 (7,1%) | 27 (9,2%) | 213 (13,1%) | 621 (17,6%) | 1,380 (20,2%) | 127 (18,1%) |
| 60-64 | 337 (10,5%) | 37 (7,7%) | 34 (11,5%) | 231 (14,3%) | 586 (16,6%) | 1,344 (19,6%) | 115 (16,4%) |
| 65-69 | 279 (8,7%) | 50 (10,4%) | 35 (11,9%) | 233 (14,4%) | 404 (11,4%) | 958 (14,0%) | 85 (12,1%) |
| 70-74 | 212 (6,6%) | 43 (8,9%) | 37 (12,5%) | 143 (8,8%) | 295 (8,4%) | 595 (8,7%) | 68 (9,7%) |
| ≥75 | 586 (18,3%) | 249 (51,7%) | 107 (36,3%) | 440 (27,2%) | 703 (19,9%) | 803 (11,7%) | 132 (18,9%) |
| Total | **3,201** | **482** | **295** | **1,620** | **3,529** | **6,844** | **700** |
